# Culture medium of bone marrow-derived human mesenchymal stem cells effects lymphatic endothelial cells and tumor lymph vessel formation

**DOI:** 10.3892/ol.2015.2868

**Published:** 2015-01-12

**Authors:** JIE ZHAN, YAHONG LI, JING YU, YUANYAUN ZHAO, WENMING CAO, JIE MA, XIAOXIAN SUN, LI SUN, HUI QIAN, WEI ZHU, WENRONG XU

**Affiliations:** 1 School of Medical Science and Laboratory Medicine, Jiangsu University, Zhenjiang, Jiangsu 212013, P.R. China; 2 Center of Prenatal Diagnosis, Nanjing Maternal and Child Health Hospital Affiliated to Nanjing Medical University, Nanjing, Jisngsu 210004, P.R. China; 3 The Affiliated Hospital, Jiangsu University, Zhenjiang, Jiangsu 212002, P.R. China

**Keywords:** mesenchymal stem cell, lymph vessel, tumor growth

## Abstract

Human bone marrow mesenchymal stem cells (hBM-MSCs) favor tumor growth and metastasis *in vivo* and *in vitro*. Neovascularization is involved in several pathological conditions, including tumor growth and metastasis. Previous studies have demonstrated that human bone marrow MSC-derived conditioned medium (hBM-MSC-CM) can promote tumor growth by inducing the expression of vascular epidermal growth factor (VEGF) in tumor cells. However, the effect of BM-MSCs on tumor lymph vessel formation has yet to be elucidated. In the present study, the effect of BM-MSCs on processes involved in lymph vessel formation, including tube formation, migration and proliferation, was investigated in human-derived lymphatic endothelial cells (HDLECs). It was identified that hBM-MSC-CM promoted the tube formation and migration of HDLECs. In addition, tumor cells were revealed to participate in lymph vessel formation. In the present study, the SGC-7901, HGC-27 and GFP-MCF-7 cell lines were treated with hBM-MSC-CM. The results demonstrated that the expression of the lymph-associated markers, prospero homeobox protein 1 and VEGF receptor-3, were increased in the SGC-7901 and HGC-27 cell lines, but not in the GFP-MCF-7 cells. The tube formation assay demonstrated that the HGC-27 cells treated with hBM-MSC-CM for 20 days underwent tube formation. These findings indicate that hBM-MSC-CM can promote tube formation in HDLECs and HGC-27 cells, which may be associated with lymph vessel formation during tumor growth and metastasis.

## Introduction

Mesenchymal stem cells (MSCs) are multipotent cells that originate from bone marrow or other tissues. MSCs are distributed almost ubiquitously between the perivascular niches of a number of human tissues and organs, and are important components in angiogenesis, local tissue repair and concomitant immunomodulation (1). In addition, MSCs can be recruited to a variety of tumors, including breast (2) and gastric cancer (3). A previous study by El-Haibi *et al* (4) reported that MSCs migrated to the site of tumorigenesis, where the MSCs were activated by cancer cells, and in turn promoted metastasis. Chaturvedi *et al* (2) revealed that hypoxia-inducible factors mediated the interactions of MSCs with breast cancer cells to promote metastasis. Furthermore, a study by Zhang *et al* (5) identified that MSCs were able to promote CXC chemokine receptor (CXCR) type 4-mediated osteosarcoma growth and pulmonary metastasis by upregulating the expression of vascular epidermal growth factor (VEGF). The CXC chemokine ligand 16 and CXCR6 signaling pathway stimulates the conversion of MSCs into cancer-associated fibroblasts, which ultimately facilitates prostate tumor metastasis (6). However, as lymphatic vessels are the main route of tumor metastasis, the changes that occur in MSCs to promote tumor metastasis have yet to be elucidated. Lymph vessels are an important component in lymph node metastasis, but the association between MSCs and lymph vessels remains unknown.

Since VEGF receptor (VEGFR)-3 was identified as the first lymphatic marker almost 20 years ago, the mechanisms underlying lymphangiogenesis and metastasis have been extensively investigated (7–9). Although it is known that blood and lymphatic vessels are the major routes of metastatic spread, cancer cells were first identified to be disseminated to lymphatic vessels rather than blood vessels in a number of cancers, including breast, colon, prostate and lung cancers, and melanoma (10). In addition, the tumor microenvironment has been demonstrated to induce the expression of lymphangiogenic factors that promote metastasis (11). Breast cancer metastasis to regional lymph nodes has been revealed to be associated with lymphatic vessel density (LVD) rather than tumor size (12). At present, the VEGF-C/VEGFR-3 signaling pathway has been indentified to be involved in lymphatic metastasis (12–16). VEGF-C is able to promote tumor lymphangiogenesis and metastasis by binding to its corresponding receptor, VEGFR-3 (13).

Cancer stem cells (CSCs), also termed tumor-initiating cells, are important for the initiation of tumorigenesis and metastasis, a phenotype that can be partly maintained by altered c-Jun N-terminal kinase signaling. CSCs subsequently affect tumorigenesis and lymphatic metastasis (10,17). In the present study, cancer cells were treated with human bone marrow MSC-conditioned medium (hBM-MSC-CM) for a period of time to allow the tumor cells to express the lymphatic vessel-associated markers Prox-1 and VEGFR-3.

## Materials and methods

### Cell culture

The primary lymphatic endothelial cells (LECs) were purchased from ScienCell (Carlsbad, CA, USA). The hMSCs were isolated, cultured and characterized as previously described (16). The human gastric carcinoma SGC-7901 and HGC-27 cell lines were purchased from the Chinese Academy of Sciences Type Culture Collection Committee cell bank (Beijing, China). The cell lines were cultured in Dulbecco’s modified Eagle’s medium (DMEM; Gibco Life Technologies, Carlsbad, CA) supplemented with 10% fetal bovine serum (FBS; Gibco) at 37°C in 5% CO_2_.

### Preparation of the hBM-MSC-conditioned medium and co-culture with tumor cells

The hMSCs were cultured to ~70% confluency, and the 5 ml of medium was refreshed prior to the cells being incubated for an additional 48 h. In total, 0.22 μm filter-sterilized supernatant was collected and designated as hBM-MSC-CM. For the pre-treatment of tumor cells with hBM-MSC-CM, the HGC-27 cells were washed three times with phosphate-buffered saline, and then incubated with hBM-MSC-CM at 37°C in 5% CO_2_ for a further five days, prior to being collected for use in the subsequent experiments.

### Tube formation assay

To perform the tube formation assay, 50 μl (10 mg/ml) growth factor-reduced Matrigel (BD Biosciences, Billerica, MA, USA) was first used to pre-coat a 96-well plate. Next, 1×10^4^ LECs or 1×10^4^ BM-MSC-CM-treated HGC-27 cells in 150 μl DMEM supplemented with 10% FBS, or one-half DMEM supplemented with 10% FBS and one-half hBM-MSC-CM medium were seeded into each well. Following 16-h incubation at 37°C, an inverted phase-contrast microscope (magnification, ×100; Eclipse Ti-S; Nikon Corporation, Tokyo, Japan) was used to observe and capture images of the tube structures. The average of two fields was taken as the value for each treatment.

### Transwell migration assay

For the Transwell migration assay, 5×10^4^ LECs/well were plated in the upper wells, which were filled with 200 μl DMEM supplemented with 1% FBS. In the lower chamber, hBM-MSC-CM was used as a chemoattractant to encourage cellular migration. The cells were incubated for 8 h at 37°C, and 10% FBS served as the control. The cells that did not migrate were removed using a cotton swab. The cells that did migrate were stained using crystal violet stain, and then counted under a microscope (Ti-S; Nikon Corporation). In total, three views were chosen at random, and each experiment was repeated independently in triplicate.

### Scratch-wound assay

The cells were seeded a density of 2×10^5^ cells/well into six-well plates (Corning Inc., Corning, NY, USA), and then cultured for ~48 h, at which time the cells had reached ~80% confluency. Subsequent to the cell monolayer being scratched with a sterile 200 μl pipette tip, the cells were treated with 0, 50, 75 or 100% hBM-MSC-CM, and then incubated for a further 12 h to allow time for migration into the cell-free area.

### MTT assay

The cell viability was determined using an MTT assay. First, the LECs were seeded into 96-well plates (Corning Inc.) at a density of 5×10^3^ cells per well, and then cultured overnight. Next, various concentrations, comprising one-half hBM-MSC-CM and one-half DMEM supplemented with 10% FBS, two-thirds hBM-MSC-CM and one-third DMEM supplemented with 10% FBS or three-quarters hBM-MSC-CM and one-quarter DMEM supplemented with 10% FBS, were added to the plates. The plates were then incubated at 37°C in a 5% CO_2_ atmosphere for 24, 48 or 72 h, respectively. The untreated SGC-7901 cells served as the control. MTT dye was added to each well for the final 4 h of treatment. The reaction was terminated by the addition of dimethyl sulfoxide (Sigma-Aldrich, St. Louis, MO, USA), and the optical density (OD) was determined at 490 nm using a multiwell plate reader (FLx800; BioTek, Winooski, VT, USA). The background absorbance of the medium in the absence of cells was subtracted. All samples were assayed in triplicate, and the mean for each experiment was calculated.

### Western-blot analysis

The proteins were extracted from the whole cell lysates using cell extraction buffer (Invitrogen, Carlsbad, CA, USA) and the protein concentration was determined. In total, 20 μg of the extracted total cellular protein from each sample was separated via SDS-PAGE, and subsequently transblotted onto a polyvinylidene fluoride membrane (Millipore, Billerica, MA, USA). The blotted nitrocellulose membranes were incubated with a polyclonal primary rabbit anti-human Prox-1 (cat. no. ab38692; dilution, 1:800; Abacam, Cambridge, UK) and polyclonal rabbit anti-human VEGFR-3 (dilution, 1:200, cat. no. 21410-2, Signalway Antibody, College Park, MD, USA) antibodies, and then with a peroxidase-conjugated goat anti-mouse (dilution, 1:2,000; CW0103, CWBIO, Beijing, China) and goat anti-rabbit (dilution, 1:2,000; KC-MM-035, Kang Chen Bio-Tech, Shanghai, China) secondary antibodies. The blots were visualized using the enhanced chemiluminescent detection system (Amersham plc., Amersham, UK) and analyzed using Image-Pro Plus version 5.1 (Media Cybernetics, Inc., Rockville, MD, USA).

### Statistical analysis

The data are expressed as the mean ± standard deviation. Statistical differences were analyzed using a one-way analysis of variance, followed by Dunnett’s multiple comparison tests. P<0.05 was considered to indicate a statistically significant difference.

## Results

### hBM-MSC-CM induces tube formation in LECs and in the gastric cancer HGC-27 cell line

**Figure 1:**
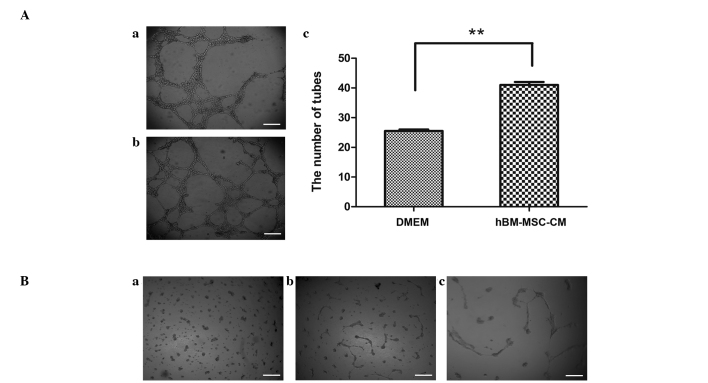
Effects of human bone marrow mesenchymal stem cell culture medium (hBM-MSC-CM) on tube formation ability. hBM-MSC-CM enhanced the tube formation ability of lymphatic endothelial cells (LECs). (A) LECs (1×10^4^/well) were incubated with either (a) Dulbecco’s modified Eagle’s medium (DMEM) supplemented with 10% fetal bovine serum (FBS) or (b) one-half DMEM supplemented with 10% FBS and one-half hBM-MSC-CM in the upper Matrigel chamber. Images of the tube-like structures were captured using a microscope (scale bar, 50μm). (c) Statistical analysis was performed using a t-test. ^**^P<0.01 compared with the DMEM group. (B) (a)Tube formation ability of HGC-27 cells, (b) hBM-MSC-CM-treated HGC-27 cells and (c) LECs.

Lymphangiogenesis is an important factor involved in neoplastic metastasis. In order to investigate the role of MSCs in lymphangiogenesis, the present study examined the effect of BM-MSCs on primary LEC tube formation. As shown in Fig. 1A and B, hBM-MSC-CM significantly induced LEC tube formation. The number of tubes in hBM-MSC-CM-treated LECs was significantly increased compared with the LECs treated with DMEM alone. These data indicate that hBM-MSC-CM may contribute to lymphangiogenesis. In addition, it was identified that the gastric cancer HGC-27 cell line treated with one-half DMEM and one-half hBM-MSC-CM for 20 days exhibited increased tube formation compared with the HGC-27 cells treated with DMEM alone (Fig. 1C).

### hBM-MSC-CM promotes lymphatic endothelial cell migration

**Figure 2:**
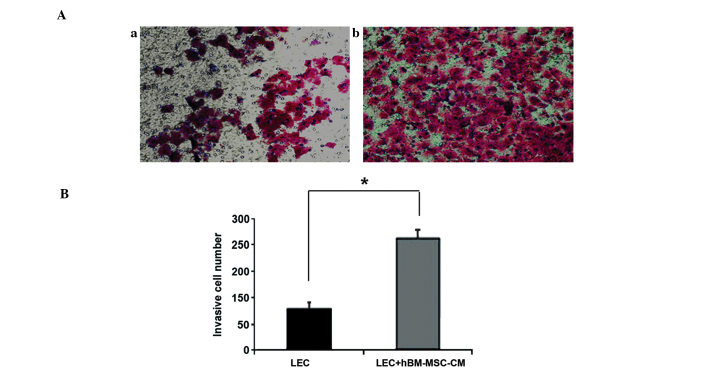
Transwell migration assay. (A) Human bone marrow mesenchymal stem cell culture medium (hBM-MSC-CM) increased the migration ability of lymphatic endothelial cells (LECs) in the (a) control and (b) 50% hBM-MSC-CM-pretreated groups (magnification, ×100). (B) The number of migrated cells in the groups was analyzed using a t-test. ^*^P<0.05 compared with the control group.

**Figure 3:**
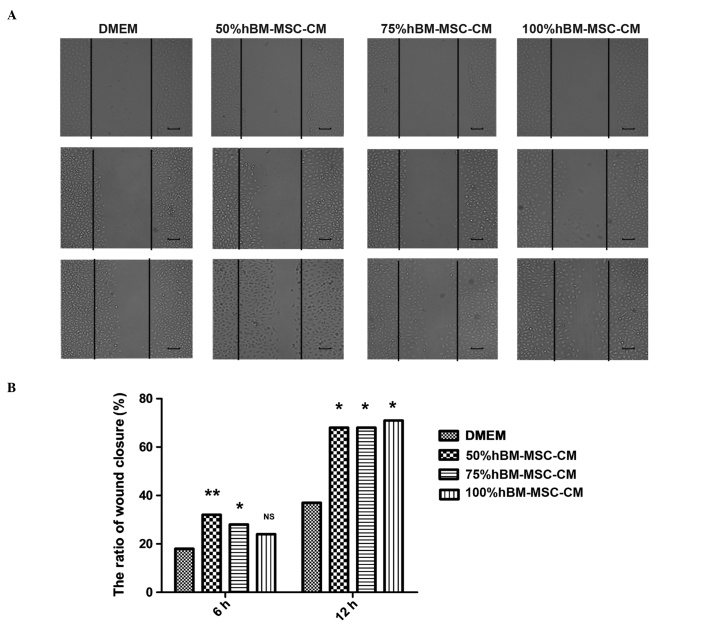
Scratch-wound assay for cellular migration. (A) Microscope analysis revealing wound closure in the cells incubated with Dulbecco’s modified Eagle’s medium (DMEM), 50% human bone marrow mesenchymal stem cell culture medium (hBM-MSC-CM), 75% hBM-MSC-CM and 100% hBM-MSC-CM (scale bar, 100 μm). (B) Ratios of wound closure in cells incubated with DMEM and 50%, 75% and 100% hBM-MSC-CM. Statistical analysis was performed by a one-way analysis of variance, followed by Dunnett’s multiple comparison tests. ^*^P<0.05 and ^**^P<0.01 compared with the DMEM control group. NS, not significant.

Cell adhesion is important for tumor lymphangiogenesis. In order to determine the effect of MSCs on the migration of LECs, the present study examined the extent of cell adhesion by performing a cell migration assay in a Transwell system. The hBM-MSC-CM-pretreated LECs exhibited a 3–4-fold increase in migration compared with those incubated with DMEM supplemented with 10% FBS alone (control group) (Fig. 2A and B), which indicated that hBM-MSC-CM promotes LEC migration. In addition, the scratch-wound assay also demonstrated that treatment with one-half DMEM and one-half hBM-MSC-CM, one-third DMEM and two-thirds hBM-MSC-CM, or hBM-MSC-CM alone demonstrates the ability to promote enhanced LEC migration compared with DMEM supplemented with 10% FBS alone (Fig. 3A and B).

### hBM-MSC-CM induces the expression of lymphatic markers in LECs, and SGC-7901 and HGC27 cells

**Figure 4:**
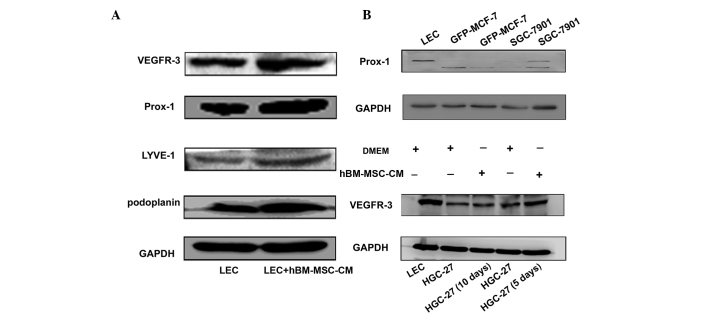
Western blot analysis revealing the effect of human bone marrow mesenchymal stem cell culture medium (hBM-MSC-CM) on lymphatic endothelial cell (LEC)-specific markers. (A) Expression of vascular epidermal growth factor receptor-3 (VEGFR-3), Prox-1, lymphatic vessel endothelial receptor-1 (LYVE-1) and podoplanin in LECs incubated for 48 h with either Dulbecco’s modified Eagle’s medium (DMEM) or hBM-MSC-CM. (B) Expression of Prox-1 in LECs and GFP-MCF-7 and SGC-7901 cells incubated with either DMEM or hBM-MSC-CM for 40 days, and the expression of VEGFR-3 in HGC-27 cells incubated with either DMEM or hBM-MSC-CM for five and 10 days.

In order to further verify the role of hBM-MSC-CM in lymphangiogenesis, the levels of the lymphatic markers podoplanin, Prox-1, VEGFR-3 and lymphatic vessel endothelial hyaluronic acid receptor-1 (LYVE-1) were analyzed in hBM-MSC-CM-treated LECs, and in SGC-7901 and HGC-27 cells. As shown in Fig. 4, high levels of podoplanin, Prox-1, VEGFR-3 and LYVE-1 were expressed in LECs following treatment with one-half hBM-MSC-CM and one-half DMEM for 48 h (Fig. 4A). Furthermore, the expression of Prox-1 in the gastric cancer SGC-7901 cell line increased following 40 days of treatment with hBM-MSC-CM (Fig. 4B). Finally, the expression of VEGFR-3 was analyzed in hBM-MSC-CM-treated HGC-27 cancer cells. The results revealed that VEGFR-3 was also upregulated in HGC-27 cells on days five and 10 after hBM-MSC-CM treatment (Fig. 4B). These results indicate that hBM-MSC-CM may promote the transition of tumor cells to LECs, and that the increased expression of Prox-1 and VEGFR-3 in cancer cells is also able to promote lymphangiogenesis. The transition to LECs induces lymphangiogenesis and tumor metastasis.

### hBM-MSC-CM demonstrated no effect on the proliferation of primary LECs

**Figure 5:**
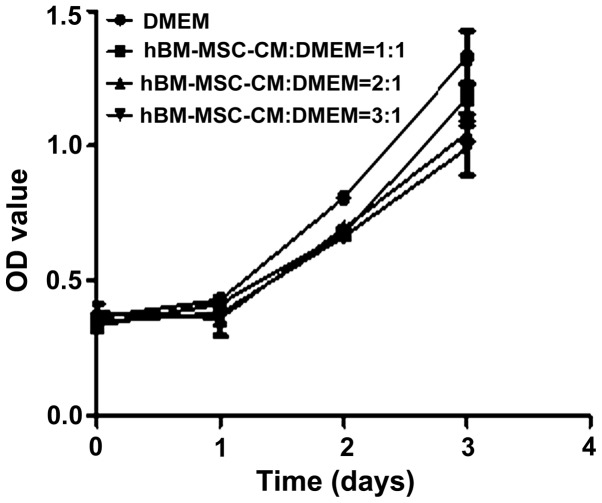
MTT assay analysis revealing the proliferation of lymphatic endothelial cells (LECs) treated with various concentrations of human bone marrow mesynchymal stem cell culture medium (hBM-MSC-CM) and Dulbecco’s modified Eagle’s medium (DMEM). The concentration of hBM-MSC-CM did not effect the proliferation of primary LECs compared with the control. hBM, human bone marrow; OD, optical density.

In addition to cell migration, cell proliferation also performs a significant role in lymphangiogenesis. Therefore, the effect of hBM-MSC-CM on LEC proliferation was investigated. The primary LECs were treated with hBM-MSC-CM at various concentrations. However, no significant difference in the rate of LEC proliferation was identified between the hBM-MSC-CM and DMEM treatment groups (Fig. 5).

## Discussion

Previous studies have reported that hBM-MSC-CM demonstrates a positive effect on tumor growth. It is hypothesized that hBM-MSC-CM may induce the expression of VEGF in tumor cells, and cause the activation of the ras homolog gene family, member A-guanosine triphosphate and extracellular signal-regulated kinase 1/2 signaling pathways (18). Other studies have also revealed that MSCs are able to promote tumor growth and metastasis, including in breast cancer (19–22), and that MSC-like cells isolated from human colon cancer tissues can increase tumor growth and metastasis (23). However, another previous study revealed that MSCs induced tumor growth in models of hepatocellular carcinoma *in vivo*, but significantly decreased the presence of lung metastases (24). Therefore, it is essential to illustrate the role of MSCs in the metastasis of SGC-7901 cells in nude mice models. In order to establish whether MSCs perform an important role in tumor metastasis, the present study treated MCF-7 and SGC-7901 cells with hBM-MSC-CM for 40 days, and then analyzed the expression of the lymphatic vessel-associated marker Prox-1 using western blot analysis. The results revealed that the SGC-7901 cells treated with hBM-MSC-CM exhibited a high expression of Prox-1. In addition, the HGC-27 cells were treated with hBM-MSC-CM, and protein samples were collected every five days. The results demonstrated that the expression of VEGFR-3 increased over 10 days. Therefore, it was hypothesized that hBM-MSC-CM contains cytokines that induce the transition of cancer cells to cells with a lymphatic phenotype, which in turn promotes tumor metastasis. Furthermore, the data revealed that hBM-MSC-CM promotes tube formation and the migration of LECs, but exerts no positive effect upon the proliferation of LECs. In future studies, it may be of interest to identify the mechanism by which MSCs promote tumor lymph vessel formation.
